# Thirty Years of Forest Census at Barro Colorado and the Importance of Immigration in Maintaining Diversity

**DOI:** 10.1371/journal.pone.0049826

**Published:** 2012-11-30

**Authors:** Richard Condit, Ryan A. Chisholm, Stephen P. Hubbell

**Affiliations:** 1 Smithsonian Tropical Research Institute, Panama City, Panama; 2 Deparment of Ecology and Evolutionary Biology, University of California, Los Angeles, Los Angeles, California, United States of America; The Pennsylvania State University, United States of America

## Abstract

The neutral theory of community ecology can predict diversity and abundances of tropical trees, but only under the assumption of steady input of new species into the community. Without input, diversity of a neutral community collapses, so the theory's predictions are not relevant unless novel species evolve or immigrate. We derive analytically the species input needed to maintain a target tree diversity, and find that a rate close to 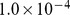 per recruit would maintain the observed diversity of 291 species in the Barro Colorado 50-ha tree plot in Panama. We then measured the rate empirically by comparing species present in one complete enumeration of the plot to those present five years later. Over six census intervals, the observed rate of input was 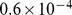 to 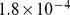 species per recruit, suggesting that there is adequate immigration of novel species to maintain diversity. Species interactions, niche partitioning, or density-dependence, while they may be present, do not appear to enhance tree species richness at Barro Colorado.

## Introduction

The crucial assertion of the neutral theory of community ecology is that diversity can be maintained in the absence of species differences as long as there is steady input of new species via speciation or immigration [1], [2]. More broadly, diversity can be maintained independent of niche divergence, or in the face of competitive dominance, given sufficient dispersal [3]–[5]. Various models produce predictions of species abundance distributions as they depend on dispersal, and these have been tested against real forests [6]–[9], but the theory [1] also includes a quantitative prediction of diversity as a function of speciation [10]. None of the previous empirical tests of neutral theory, however, considered the speciation parameter. In the absence of novel species input, the neutral theory is irrelevant, and stabilizing mechanisms such as niche differentiation among species or competitive interactions must maintain diversity [11], [12].

Here we make use of repeated censuses of the Barro Colorado 50-ha forest plot in Panama to examine the rate at which novel species appear; we call this the rate of *species input*. There have been seven complete censuses over 30 years, and each of the last six provides a direct estimate of the rate of input. The simple and obvious test is whether there has been any at all: have any novel species recruited into the 50 hectares since the initial census of 296 species in 1982 [13]? The flora of Barro Colorado Island is well known, and we would certainly know new species. The more precise test is whether the observed species input is high enough to maintain the plot's observed diversity. The theory provides an exact prediction about what would be sufficient.

Our aim does not end with a qualitative confirmation or rejection. We intend to measure a rate constant that is relevant in the ecological theory that accommodates stochastic births and deaths, dispersal, and species interactions [14]–[20]. Whatever value we find, the rate at which new species immigrate leads to inferences about the forces that are key in maintaining species diversity.

## Materials and Methods

### Theory

In the basic neutral theory, a community of 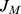 individuals is subject to random deaths and births. At each time step, one individual dies, with every individual equally likely, and then one of the survivors is chosen at random to become the parent of a replacement. At a constant rate 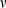, the newborn mutates and becomes a new species, hence 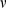 is called the speciation rate [1] and is equal to the probability that any newborn is a novel species. If 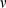 is constant, then species diversity and the full species abundance distribution eventually reach a dynamic equilibrium around which they will subsequently fluctuate randomly. The equilibrium can be derived analytically, and a single parameter, 
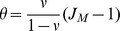
, the biodiversity parameter, fully describes it [1], [6]. 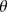 turns out to be asymptotically equal to Fisher's diversity statistic 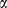, defined by
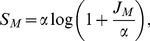
(1)where 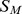 is the number of species in the community. Setting 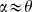, this leads to a prediction about the speciation rate that would maintain 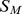 species in the community,
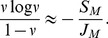
(2)


This formulation holds only for a *metacommunity*: a community into which there is no immigration and within which there is unlimited dispersal, meaning every individual is equally likely to be the parent of any birth. To relax both assumptions and accommodate limited dispersal, consider a small subset of the metacommunity termed the *local community*. Any subset will do, as long as the boundaries are unvarying so that immigration of newborn from outside has a consistent meaning: offspring whose parents reside outside the local community. We imagine the metacommunity as a continent of trees and the local community as a rectangular plot with precise but unchanging borders, but the theory accommodates more general arrangements. Define the migration rate *m* as the proportion of births in the local community whose parents are outside [1], or equivalently the probability that a newborn comes from outside. The remaining 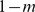 births are from local parents. Immigration alters the local species abundance distribution, and Equations 1–2 no longer hold. There are various derivations of the abundance distribution in a local community, providing estimators of both 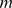 and 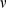 [8], [10], [21].

Still however, 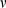 refers to speciation in the entire community, and we need to know it for the local community, where both genetic variants causing true speciation and arrival of novel species via immigration must be considered. The former is simply 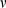, but we need a derivation for the latter: the probability 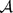 that a recruit in the local community is the immigrant offspring of a novel species from outside. 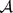 is the ratio of new species to all immigrants, equal to the probability that a randomly chosen immigrant is a species not present locally. We assume genetic speciation is very rare locally, so 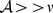, and henceforth consider 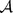 as the only species input parameter relevant to diversity in a small community.

To find 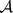, first define 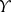 as the probability that an individual randomly selected from outside the local community is a novel species, meaning it belongs to a species not currently in the local community. Then we can write 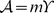, because to be a novel species, a recruit must be an immigrant (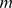), and the immigrant must be a new species (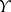). We first derive an explicit formula for 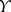 using a previously published formula [21] for the probability of any local abundance given the metacommunity abundance (Appendix S1). The leads to an expression for 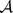 as a function of 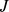 and 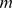:
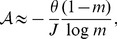
(3)where 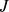 is the local community size and 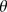 the biodiversity parameter defined above. Using Equation 7 in [10], we can remove 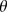 from the formula, using instead 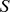, the number of species in the local community. Then
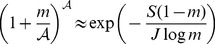
(4)and
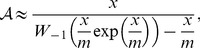
(5)where 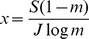 and 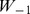 is the lower branch of Lambert's *W* function (Appendix S1). Equation 5 parallels Equation 2 as a way of estimating species input given species richness and community size, but with migration rate 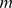 also needed because it is a local community. It turns out, however, that 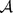 is only weakly dependent on *m* (Appendix S1), which has an intuitive interpretation: larger *m* means more births are from parents outside the local community, however, it also means that fewer species are absent from the local community.

In the Barro Colorado 50-ha tree census (see Methods), there were 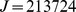 individuals and 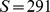 species in 1990. Using 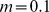, we find 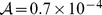. This value of 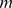 has been derived several times by various means [1], [10], [22], but always based on trees 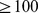 mm. Using the program Tetame (http://www.edb.ups-tlse.fr/equipe1/chave/tetame.htm), we applied the formulation from [22] to arrive at 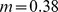 for trees 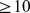 mm. This leads to 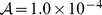, meaning that a near four-fold increase in *m* leads to a 50% increase in 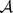. Indeed, from 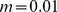 to 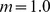, 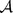 varies only three-fold (Appendix S1).

Hence, if the Barro Colorado forest were a community of fixed size, all species were demographically identical, 38% of recruits came from parents outside, and one of every 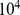 recruits was a novel species, the observed equilibrium diversity would be 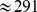 species. Our question is simply whether this predicted rate of species input is in fact observed. If it is, then local diversity can be attributed to species input, and no local diversifying mechanisms are needed. If we do not observe species input, then we must look to local processes for the maintenance of diversity.

## Methods

### Ethics statement

All research was conducted at the Barro Colorado Nature Monument, a forest preserve owned by the nation of Panama and managed by the Smithsonian solely for scientific studies and protected from all other uses. A long-term agreement with the Government of Panama assures the Smithsonian permission to continue the research indefinitely. No protected species were sampled during the forest census.

### Plot census

Since 1982, a rectangle of forest 1000 m×500 m on Barro Colorado Island, Panama, whose southwest corner is at 9.15125°N. latitude, 79.85530°W. longitude, has been fully enumerated seven times. During the initial survey (1981–1983), every individual 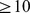 mm in stem diameter was given a numbered aluminum tag and measured; in 1985 and every 5 years since, tagged trees were remeasured or noted as dead, and new trees 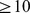 mm diameter were given tags [23], [24]. Trees were identified to species by three experts, and voucher specimens were collected and deposited in two herbaria in Panama (STRI, PMA).

Since our results hinge on population changes in rare species, we considered carefully how misidentification would affect the calculations. In a random sample of re-identified trees, we found 0.85% misidentified (159 of 18694). In the rarest species – the 44 having 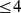 individuals (in 1990) – we double-checked every individual (in 1995), and found just one out of 85 individuals was misidentified, a *Hamelia axillaris* mistakenly called the rare *H. patens* [24]. Other *H. patens* were correctly identified, and in no case did a misidentification either remove the last record of a species or create a novel species. There were four rare taxa that we omitted from calculations due to taxonomic uncertainty: one unidentified tree in the genus *Nectandra*, possibly a novel species but never seen flowering before its death; a single tree in the genus *Apeiba* that could not be identified before it died (it appeared to be hybrid between the two well-known species); and both species in the genus *Trema*, originally identified as one species but later separated (we re-identified every living individual after 2000, but several trees that died earlier remain forever unidentified). The remaining species subject to extinction or invasion are very well known to us; most of them occur in our tree plots elsewhere in Panama [25], and we have observed every one of them outside the 50-ha plot. We are thus confident that our estimate of species turnover is based on true extinction and invasion, and that it is unbiased, since misidentifications could cause either errors of omission (misidentification of a rare species as a common species and thus failure to detect invasion or extinction) as well as commission (misidentification of a common species as a rare species and thus an apparent case of invasion or extinction when there was none).

Criteria for including stems in the census were applied consistently and define the local community of our theory: free-standing, woody stems at least 10 mm in diameter. Species known to be lianas at maturity were never included. On the other hand, individuals of species known to be stranglers (hemiepiphytes) at least some of the time were tagged whenever they were free-standing. To be consistent with the liana method, we excluded all stranglers from analyses here (nine *Ficus* and one *Oreopanax* species). Reinserting them in the calculations had a trivial impact on the final estimates.

Recruits were defined as newly appearing stems, those growing from 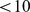 mm stem diameter in one census to 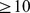 mm in the next [26]. Deaths were trees with stems 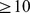 mm in one census but with no such stems alive in the next census, meaning we considered a tree ‘dead’ even if it maintained a living base [27]. This definition is required to guarantee book-keeping of the population 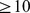 mm: adding recruits and subtracting deaths is how populations changed.

### Observed recruitment and species input

Following the theory, we define a species input event as any case where a species absent from the plot in one census appeared in the next, and an extinction event as the opposite. An intuitive estimate of the rate of species input between any pair of censuses is the number of novel species divided by the number of recruits. Likewise, the extinction rate can be simply defined as the number of extinctions divided by the number of deaths. But recruitment, input, death, and extinction are continuous processes, and a more precise estimate can be generated by solving differential equations describing their rates (Appendix S2). The estimated input rate based on the continuous solution differs only slightly from the intuitive estimate because the rates are low.

Cases where species became locally extinct in one census interval, then reappeared in a later census, were counted once as extinction and later as species input. Such input does indeed maintain diversity. Hypothetically, there might be a time far in the future when all species in the region have passed through the 50 hectares at least once, when every input event would be a species that had already been in the plot. This would still comprise a community in which local diversity is controlled by the rate of species input from outside [28].

## Results

In every census interval, there was species input and extinction (Table 1). A total of 308 non-strangler species 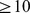 mm stem diameter were observed in the 50-ha plot during at least one of the seven censuses, but only 275 species were present in all seven censuses. The other 33 species had some turnover: they were absent in at least one census (Table 2).

**Table 1:** Rates of species turnover during six census intervals in the Barro Colorado 50-ha plot.

	Number of individuals			Number of species			Rate ( 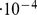 )	
Interval	Initial	Dead	Recruited	Initial	Input	Extinct	Input ( 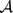 )	Extinction ( 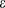 )
1982–1985	235256	26330	33073	296	2	1	0.57	0.35
1985–1990	241999	37404	39377	297	4	7	0.94	1.72
1990–1995	243972	36750	21747	294	4	5	1.70	1.30
1995–2000	228969	36703	21458	293	4	6	1.71	1.55
2000–2005	213724	31422	26035	291	5	5	1.77	1.49
2005–2010	208337	30405	29243	291	3	6	0.95	1.83

The initial number (individuals or species) is the number at the start of the census interval; the other columns all refer to change across the intervals: deaths, recruits, input (number of novel species), and extinctions (locally extinct from the plot). The calculations of the rate constants are based on formulae given in Appendix S2.

**Table 2:** Abundance (number of individuals 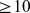 mm stem diameter) in each of the seven censuses, 1982–2010, of the 33 species in the Barro Colorado 50-ha plot that were absent in at least one census, of the total of 308 species observed in the plot.

Species	1982	1985	1990	1995	2000	2005	2010	Trees	Dbh
*Annona hayesii*	1	1	1	1	0	0	0	146	117
*Bactris coloradonis*	38	17	6	2	0	0	0	165	82
*Banara guianensis*	0	0	4	5	1	0	0	7	140
*Bertiera guianensis*	2	2	1	1	0	0	0	5	20
*Cecropia longipes*	0	0	0	1	12	14	13	48	298
*Clidemia septuplinervia*	1	2	0	0	1	3	0	7	13
*Cyathea petiolata*	8	3	1	0	0	0	0	16	160
*Geonoma interrupta*	19	14	3	0	0	0	0	107	63
*Hamelia patens*	0	2	2	1	1	1	0	4	40
*Inga mucuna*	1	1	1	1	3	1	0	4	143
*Koanophyllon wetmorei*	15	12	12	9	3	0	0	21	79
*Leandra dichotoma*	0	1	1	0	1	1	1	7	15
*Lycianthes maxonii*	1	1	0	0	0	0	0	1	10
*Miconia dorsiloba*	0	0	0	0	1	2	2	2	33
*Miconia prasina*	0	0	2	2	2	2	4	41	169
*Pavonia dasypetala*	1	1	0	0	0	0	0	8	60
*Piper imperialis*	9	3	3	1	1	0	0	9	60
*Psychotria brachiata*	0	0	1	1	0	1	3	14	31
*Psychotria hoffmannseggiana*	5	1	2	0	0	0	1	9	20
*Psychotria psychotriifolia*	0	0	0	1	1	1	0	1	18
*Psychotria racemosa*	1	2	2	0	0	2	7	13	19
*Psychotria tenuifolia*	7	5	4	1	0	2	0	15	20
*Rauvolfia littoralis*	0	0	0	1	1	1	1	13	302
*Schefflera morototoni*	1	1	0	0	0	0	0	134	631
*Solanum arboreum*	1	1	0	0	0	0	0	2	15
*Solanum asperum*	0	0	4	5	8	7	8	24	83
*Solanum circinatum*	5	4	3	1	0	5	5	23	56
*Stemmadenia grandiflora*	1	1	0	0	0	1	0	20	188
*Ternstroemia tepezapote*	1	1	1	1	1	0	0	8	650
*Vasconcellea cauliflora*	0	0	0	0	0	0	2	7	182
*Verbesina gigantea*	0	0	0	0	1	1	2	4	41
*Vismia macrophylla*	1	0	0	1	1	0	2	102	353
*Xylosma chlorantha*	1	1	0	0	0	0	0	1	20

Each species' abundance in 55 plots within 35 km of Barro Colorado [25] is included (under Trees), showing that 29 of the 33 species are known to us elsewhere. Dbh shows the maximum stem diameter (mm) across the same 55 plots.

For example, in 2010 there were three new arrivals relative to 2005: *Vasconcellea cauliflora*, *Vismia macrophylla*, and *Psychotria hoffmannseggiana* (Table 2). *Vasconcellea* is rare throughout the region but unmistakable even to novices, having large, extremely lobed leaves like those of the related papaya (*Carica papaya*). Prior to 2010, we had never seen it in the 50-ha plot. *Vismia* is also easy to recognize, since it is abundant along roadsides of wet and submontane forest nearby, but at Barro Colorado it is rare. It was found in the plot prior to 2010, but went extinct (twice) and has now (twice) re-invaded (Table 2). The final invader, *P. hoffmannseggiana*, is a rare shrub that can only be identified by experts; it also went extinct then reinvaded. Those three immigrant species were found among 29243 recruits since 2005, thus the intuitive input rate per recruit 
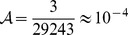
. The dynamic rate estimate was slightly lower, 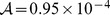 (Table 1).

Over six census intervals, 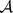 varied from 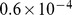 to 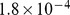 new species per recruit (Table 1), while according to theory, 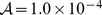 would maintain 291 species in the 50-ha plot. That assumes a migration parameter of 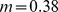, but with the lower migration rate, 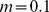, the input rate needed according to theory would be only slight lower, 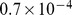. The observed rate was close, falling within the theoretical range twice and barely lower once (Table 1). When differing most, the observed rate was higher than predicted, though by less than threefold.

## Discussion

Tree species have been continually input into the 50-ha plot at Barro Colorado over 30 years, and the observed rate was consistent through time and a quantitative match to the theoretical rate needed to maintain diversity. There is nothing circular in our estimates of observed and predicted input: the theoretical rate depends on local species richness (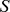), community size (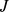), and immigration (*m*), none of which depends on novel species [22]. Indeed, given these values of 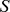, 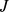, and *m*, an observation of no new species and thus zero input was completely plausible.

We have known since the first census that the 50-hectare plot is a subset of a regional community, because Croat's [29] flora of Barro Colorado Island includes 450 tree and treelet species, leaving nearly 150 species absent from the plot [30]. A few of those are specialists in habitats not found within the 50 hectares, such as the pond apple (*Annona glabra*) of the lake shore, but most are upland species that could grow in the plot. Future censuses will capture more of those species, while others will continue to drop out. The 2010 census included 13 singletons (species with a single individual), and these are at obvious risk of local extinction: 10 of the 17 singletons in 1982 are now extinct. But it is not just singletons subject to turnover. Three species with 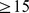 individuals in 1982 are now extinct, and *Cecropia longipes*, which invaded the plot in 1995, now has 13 individuals.

Had we observed a rate of species input substantially lower than the prediction for maintaining diversity we would have concluded that stabilizing mechanisms, i.e. rare species advantage, competition, or niche differentiation [11], are important in maintaining species richness. Had the rate been too high, we would have been forced to consider destabilizing mechanisms that drive rare species to extinction faster than expected by chance. We conclude instead that species input is maintaining tree richness in the Barro Colorado plot. Stabilizing forces may be present, and they may limit abundances [31], but they do not contribute to diversity. Indeed, the observed extinctions demonstrate that whatever stabilizing forces are present are insufficient to protect rare species. These conclusions conform with a variety of theoretical studies showing that dispersal can overwhelm niche differences as the driver of diversity and community structure [4], [14], [32], so that regional diversity can regulate local diversity [28], [33]–[37].

The importance of species input explains the success of the neutral model in predicting abundances in spite of evident non-neutrality [38]–[40]. When species input dominates, abundances resemble the neutral prediction, particularly in the long tail of rare species, even if there are species differences [5], [14]. In fact, the zero-sum multinomial abundance distribution predicted by the neutral theory generalizes to habitat-partitioned communities as long as there is species input [17], [41]. The neutral model predicts diversity and abundance at Barro Colorado because it properly describes what matters most – species input – while ignoring irrelevant details [32], [38], [41]. The theory also predicts exactly how much species input is sufficient to maintain richness, and that when insufficient, diversifying mechanisms spawned by species differences must account for abundances and diversity.

Diversifying mechanisms at wider scales are not addressed by these results. There is a regional species pool from which the 50-ha plot is drawing, and there may be niche differentiation maintaining diversity in the wider region. The role of species input (speciation of any kind) in maintaining diversity at larger scales remains untested, because precise measures of species interactions and species input are not now possible beyond local plots. The rate of immigration of novel species must decline as area increases, but the rate of input needed to maintain diversity also declines. Perhaps over the entire nation of Panama or the continent of South America, speciation is too rare to maintain diversity without niche segregation [42], or perhaps species input via true genetic speciation is what drives diversity and abundances at continental scales.

## Supporting Information

Appendix S1 **Arrival rate of new species in a local community of Hubbell's spatially implicit model.**
(PDF)

Appendix S2 **Estimating rates of species extinction and input.**
(PDF)
