# Supplementary material for: Thirty Years of Forest Census at Barro Colorado and the Importance of Immigration in Maintaining Diversity
Source: PLoS One. 2012 Nov 30;7(11):e49826. doi: 10.1371/journal.pone.0049826 (PMC3511510; doi:10.1371/journal.pone.0049826)
Supplement: Appendix S1 — Arrival rate of new species in a local community of Hubbell's spatially implicit model. (PDF) [file pone.0049826.s001.pdf]

Thirty years of forest census at Barro Colorado and the importance of immigration in maintaining diversity: Appendix S1

Richard Condit[1,*], Ryan A. Chisholm[2], Stephen P. Hubbell[3]

1 Smithsonian Tropical Research Institute, Panama

2 Smithsonian Tropical Research Institute, Panama

3 Smithsonian Tropical Research Institute, Panama, & Deparment of Ecology and Evolutionary Biology, University of California, Los Angeles, USA

∗ **E-mail: conditr@gmail.com**

Appendix S1: Arrival rate of new species in a local community of Hubbell's spatially implicit model

We seek the theoretical rate of arrival of new species into a local community that would account for observed species diversity in Hubbell's spatially implicit neutral model [1]. We define the arrival rate, $\mathcal{A}$, as the proportion of recruitment events inside the local community that result in a new species (i.e., a species not present in the local community at the time) due to migration. A second potential source of new species would be novel genetic combinations [2], but we make the usual simplifying assumption that the latter is so rare in the local community that we can ignore it. The arrival rate of new species per recruit is then the product of two probabilities: 1) that a recruit is an immigrant from the metacommunity (m) and 2) that a randomly selected individual from the metacommunity is a species not currently present in the local community (Υ).

We use the following standard definitions: J is the (constant) local community size;

J_M is the (constant) metacommunity size; m is the immigration parameter; and ν is the speciation rate in the metacommunity. We also define the composite parameters $\theta = (J_M - 1)\frac{\nu}{1-\nu}$ (the fundamental biodiversity number) and $\lambda = (J-1)\frac{m}{1-m}$. We seek an expression for the arrival rate $\mathcal{A}$ of new species into the local community.

The probability that a species has abundance n in the local community given that it has relative abundance x in the metacommunity is [3]

$$P_S\,(n; J, m, x) = \binom{J}{n} \frac{\Gamma(\lambda)}{\Gamma(J+\lambda)} \frac{\Gamma(J-n+\lambda-\lambda x)}{\Gamma(\lambda-\lambda x)} \frac{\Gamma(n+\lambda x)}{\Gamma(\lambda x)}. \tag{1}$$

The probability that a species has abundance n in the metacommunity is

$$\langle \Phi_n \rangle_M = \frac{\theta}{n} \frac{\Gamma(J_M+1)}{\Gamma(J_M+1-n)} \frac{\Gamma(J_M+\theta-n)}{\Gamma(J_M+\theta)}. \tag{2}$$

Thus, the probability Υ that a randomly selected individual in the metacommunity belongs to a species with abundance zero in the local community is

$$\begin{aligned}
\Upsilon &= \sum_{n=1}^{J_M} \frac{n \langle \Phi_n \rangle_M}{J_M} P_S\left(0; J, m, \frac{n}{J_M}\right) \\
&= \sum_{n=1}^{J_M} \frac{\theta}{J_M} \frac{\Gamma(J_M+1)}{\Gamma(J_M+1-n)} \frac{\Gamma(J_M+\theta-n)}{\Gamma(J_M+\theta)} \frac{\Gamma(\lambda)}{\Gamma(J+\lambda)} \frac{\Gamma\left(J+\lambda-\lambda\frac{n}{J_M}\right)}{\Gamma\left(\lambda-\lambda\frac{n}{J_M}\right)} \\
&\approx \frac{\theta}{J_M} \sum_{n=1}^{J_M} (1-\nu)^n m^{\lambda\frac{n}{J_M}} \\
&\approx \theta \int_{x=0}^{\infty} (1-\nu)^{xJ_M} m^{\lambda x} dx = -\frac{\theta}{\lambda \log m + J_M \log(1-\nu)} \\
&\approx -\frac{\theta}{\lambda}\frac{1}{\log m}.
\end{aligned} \tag{3}$$

The approximation to the summand is good for small values of n and goes to zero for

large values of n, consistent with the behavior of the original summand. The replacement of the summation by the integral to infinity is a valid approximation because J_M is large and because the summand decays rapidly as n increases.

The arrival rate of new species is then obtained by multiplying the above result by m:

$$\mathcal{A} \approx -\frac{\theta}{\lambda}\frac{m}{\log m} = -\frac{\theta}{J}\frac{(1-m)}{\log m}. \tag{4}$$

We can express $\mathcal{A}$ in terms of the species richness in the local community S instead of the fundamental biodiversity number θ by using an expression for S from [4]:

$$S \approx \theta \log\left(1 - \frac{\lambda}{\theta}\log m\right) \tag{5}$$

Combining with our expression for $\mathcal{A}$ and rearranging gives

$$\left(1 + \frac{m}{\mathcal{A}}\right)^{\mathcal{A}} \approx \exp\left(-\frac{S(1-m)}{J\log m}\right), \tag{6}$$

and solving for $\mathcal{A}$ produces our final formula

$$\mathcal{A} \approx \frac{x}{W_{-1}\left(\frac{x}{m}\exp\left(\frac{x}{m}\right)\right) - \frac{x}{m}}, \tag{7}$$

where $x = \frac{S(1-m)}{J\log m}$ and W_{-1} is the lower branch of Lambert's W function.

Using the empirical values $S = 291$ and $J = 213724$ from the BCI plot, the value of $\mathcal{A}$ ranges from 0.000056 (for $m = 0.01$) to 0.000155 (for $m = 1.0$). This means that $\mathcal{A}$ is close to $1 \cdot 10^{-4}$ over the entire plausible range of m.

References

1. Hubbell SP (2001) *The Unified Neutral Theory of Biodiversity and Biogeography.* NJ, Princeton University Press: Princeton.

2. Melián CJ, Alonso D, Allesina S, Condit RS, Etienne RS (2012) Does sex speed up evolutionary rate and increase biodiversity? PLoS Computational Biology 8: e1002414.

3. Alonso D, McKane AJ (2004) Sampling Hubbell's neutral theory of biodiversity. Ecology Letters 7: 901-910.

4. Volkov I, Banavar JR, Hubbell SP, Maritan A (2007) Patterns of relative species abundance in rainforests and coral reefs. Nature 450: 45-49.
