# Supplementary material for: Thirty Years of Forest Census at Barro Colorado and the Importance of Immigration in Maintaining Diversity
Source: PLoS One. 2012 Nov 30;7(11):e49826. doi: 10.1371/journal.pone.0049826 (PMC3511510; doi:10.1371/journal.pone.0049826)
Supplement: Appendix S2 — Estimating rates of species extinction and input. (PDF) [file pone.0049826.s002.pdf]

Thirty years of forest census at Barro Colorado and the importance of immigration in maintaining diversity: Appendix S2

Richard Condit[1,*], Ryan A. Chisholm[2], Stephen P. Hubbell[3]

1 Smithsonian Tropical Research Institute, Panama

2 Smithsonian Tropical Research Institute, Panama

3 Smithsonian Tropical Research Institute, Panama, & Deparment of Ecology and Evolutionary Biology, University of California, Los Angeles, USA

∗ **E-mail: conditr@gmail.com**

Appendix S2: Estimating rates of species extinction and input

We derive here empirical estimates for rates of species input and extinction in a dynamic population model, given censuses at two discrete times. We assume a population of trees composed of a mixture of species all of which are subject to equal probabilities of recruitment and death. Recruitment is defined as the number of new recruits per existing individual per time, usually called the per capita recruitment rate, or equivalently, the probability that any individual produces one recruit per unit of time. The per capita death rate is the probability that an individual dies per time. Intuitively, the recruitment rate is the number of recruits at time t divided by the number of individuals alive at time 0, divided by t, but this estimate is precise only in the limit as $t \to 0$, and likewise for the death rate.

Intuitively, the observed rate of species input per recruit is the number of new species

divided by the number of recruits over the time interval, or equivalently, the probability that any recruit is a new species, and likewise for extinction. As for recruitment and death rates, however, these intuitive estimates are precise only in the limit as $t \to 0$. The purpose of the derivation below is to find exact estimates of the instantaneous rates given a finite census interval. An additional caveat with a finite census interval is that all rates may fluctuate through time, and if they do, the estimates presented below are time-averaged instantaneous rates.

Define:

$t =$ the time interval;

$S = S(t) =$ number of extant species as it varies throughout time, writing $S(0) = S_o$;

$K = K(t) =$ number of the initial species still extant at future times (so $K \leq S_o$);

$J = J(t) =$ number of individuals as it varies through time, writing $J(0) = J_o$;

$N = N(t) =$ number of survivors of the initial J_o individuals as it varies through time (so $N \leq J$);

$H = H(t)$ number of individuals of the species originally present (so H can be $> N$ because those species recruit new individuals);

$\mathcal{A} =$ rate of arrival of new species per recruit (see Appendix S1);

$\epsilon =$ rate of extinction per death;

$r =$ per capita recruitment rate;

$d =$ per capita death rate.

Start with the demographics of recruitment and death, describing change in the total number of individuals and the number of surviving individuals, leading to dynamic estimates of the rate constants for recruitment and death [1]:

$$r - d = \frac{\ln J - \ln J_o}{t}, \tag{1}$$

$$d = \frac{\ln J_o - \ln N}{t}, \tag{2}$$

$$r = \frac{\ln J - \ln N}{t}. \tag{3}$$

Next create a similar formulation for H, the number of individuals belonging to the original S_o species. H declines with death at rate d while it increases with recruitment at rate $r(1 - \mathcal{A})$. $\mathcal{A}$ must be subtracted because it refers to recruits belonging to novel species, and thus not part of H. Then

$$H = J_o e^{[r(1-\mathcal{A})-d]t} = J_o e^{\omega t}, \tag{4}$$

where $\omega = r(1 - \mathcal{A}) - d$.

Now consider extinction alone. Assume once extinct, species do not return. Then the number of surviving species declines with deaths among the H individuals of the original species, so the rate of loss of the original species is

$$\frac{dK}{dt} = -\epsilon dH = -\epsilon d J_o e^{\omega t}. \tag{5}$$

This assertion does not make sense in the long-term, if H is expanding, which is possible if $\omega > 0$. In that case, $\frac{dK}{dt} \to 0$ because the abundance of one last species $\to H$, so it could never go extinct. We assume the community is never near this extreme.

Solving the differential Equation (5) and rearranging produces an equation relating the extinction rate ϵ to observed population sizes:

$$\epsilon d = \omega \left(\frac{S_o - K}{H - J_o} \right) = [r(1 - \mathcal{A}) - d] \left(\frac{S_o - K}{H - J_o} \right). \tag{6}$$

Equation (6) also includes recruitment (r) and death (d) rates, but these are known from

Equations (2) and (3). In the special case where $H = J_o$ so $\omega = 0$, Equation (6) fails; then the differential in Equation (5) is constant, leading to an extinction rate estimate identical to the intuitive one, the number of extinctions $(S_o - K)$ divided by the number of deaths (dJ_o) per time:

$$\epsilon d = \frac{S_o - K}{J_o t}. \tag{7}$$

Equation (6) also includes the unknown rate of species input, $\mathcal{A}$, so it is not yet a solution for ϵ. To get a second relationship involving $\mathcal{A}$ and ϵ, we build a formula using the total number of species, S, instead of the surviving species, K. S changes with both recruitment (and species input per recruit) and death (and extinction per death):

$$\frac{dS}{dt} = (\mathcal{A}r - \epsilon d)\, J. \tag{8}$$

Solving the differential equation leads to

$$r\mathcal{A} = (r - d)\left(\frac{S - S_o}{J - J_o}\right) + \epsilon d. \tag{9}$$

This depends on $r \neq d$. If $r = d$, the differential in Equation (8) is constant and

$$ra = \epsilon d + \left(\frac{S - S_o}{J_o t}\right). \tag{10}$$

This is essentially the intuitive estimate for $\mathcal{A}$, the number of new species divided by births, adding extinctions.

Combining Equations (6) and (9) with Equations (1) and (2) leads, after a good deal of rearranging, to explicit solutions for ϵ and $\mathcal{A}$ in the most general case:

$$\mathcal{A} = \left(\frac{\ln J - \ln J_o}{\ln J - \ln N}\right)\left(\frac{X_1 + X_2}{1 + X_2}\right) \tag{11}$$

and

$$\epsilon = \left(\frac{\ln J - \ln J_o}{\ln J_o - \ln N}\right)\left(\frac{1 - X_1}{1 + X_2}\right) X_2, \tag{12}$$

where $X_1 = \left(\frac{S-S_o}{J-J_o}\right)$ and $X_2 = \left(\frac{S_o-K}{H-J_o}\right)$. Special cases of Equations (7) and (10) change these, but we have not encountered the special cases and omit their final solutions. Equations (11) and (12) provide estimates for the instantaneous rate constants of species input and extinction, averaged over the census time interval.

References

1. Condit R, Ashton P, Manokaran N, LaFrankie J, Hubbell S, et al. (1999) Dynamics of the forest communities at Pasoh and Barro Colorado: comparing two 50-ha plots. Philosophical Transactions of the Royal Society of London Series B: Biological Sciences 354: 1739-1748.
